# Complete mitochondrial genome of *Clistocoeloma sinensis* (Brachyura: Grapsoidea): Gene rearrangements and higher-level phylogeny of the Brachyura

**DOI:** 10.1038/s41598-017-04489-9

**Published:** 2017-06-23

**Authors:** Zhao-Zhe Xin, Yu Liu, Dai-Zhen Zhang, Xin-Yue Chai, Zheng-Fei Wang, Hua-Bin Zhang, Chun-Lin Zhou, Bo-Ping Tang, Qiu-Ning Liu

**Affiliations:** Jiangsu Key Laboratory for Bioresources of Saline Soils, Jiangsu Synthetic Innovation Center for Coastal Bio-agriculture, Jiangsu Provincial Key Laboratory of Coastal Wetland Bioresources and Environmental Protection, School of Ocean and Biological Engineering, Yancheng Teachers University, Yancheng, 224051 China

## Abstract

Deciphering the animal mitochondrial genome (mitogenome) is very important to understand their molecular evolution and phylogenetic relationships. In this study, the complete mitogenome of *Clistocoeloma sinensis* was determined. The mitogenome of *C*. *sinensis* was 15,706 bp long, and its A+T content was 75.7%. The A+T skew of the mitogenome of *C*. *sinensis* was slightly negative (−0.020). All the transfer RNA genes had the typical cloverleaf structure, except for the *trnS1* gene, which lacked a dihydroxyuridine arm. The two ribosomal RNA genes had 80.2% A+T content. The A+T-rich region spanned 684 bp. The gene order within the complete mitogenome of *C*. *sinensis* was identical to the pancrustacean ground pattern except for the translocation of *trnH*. Additionally, the gene order of *trnI*-*trnQ*-*trnM* in the pancrustacean ground pattern becomes *trnQ*-*trnI*-*trnM* in *C*. *sinensis*. Our phylogenetic analysis showed that *C*. *sinensis* and *Sesarmops sinensis* cluster together with high nodal support values, indicating that *C*. *sinensis* and *S*. *sinensis* have a sister group relationship. The results support that *C*. *sinensis* belongs to Grapsoidea, Sesarmidae. Our findings also indicate that Varunidae and Sesarmidae species share close relationships. Thus, mitogenomes are likely to be valuable tools for systematics in other groups of Crustacea.

## Introduction

Mitochondrial DNA (mtDNA) is a typically closed circular molecule approximately ranging in size from 14 to 18 kb. It contains 13 protein-coding genes (PCGs), 2 ribosomal RNA (rRNA) genes, 22 transfer RNA (tRNA) genes, and control region (CR)^1, 2^. mtDNA is characterized by maternal inheritance, simple structure, a small genome size, conserved gene content and organization, high mutation rate, and accelerated rate of nucleotide substitution^3–7^. The mitogenomes of animal mtDNA can provide important information on rearrangement laws and phylogenetic analysis because of their rapid evolutionary rate and lack of genetic recombination^1^. It is becoming increasingly common to use complete animal mitogenomes for phylogenetic reconstruction^8–10^. Partial DNA sequences are often too short to contain sufficient phylogenetic information^11^, and combination of mitochondrial and nuclear genomes makes model selection difficult^12^. Further, the addition of rRNA makes alignment ambiguous^13^.

The infraorder Brachyura contains about 7000 described species in 98 families^14^. *C*. *sinensis* is one of the most important Brachyura species, and is used as a good indicators of environmental changes and water pollutions in China^15^. Although *C*. *sinensis* was described over 80 years ago^16^, it is still very poorly understood. Earlier studies classified *C*. *sinensis* into Grapsidae, Sesarminae^17^. In recent years, some researchers have classified *C*. *sinensis* into Grapsoidea, Sesarmidae^18^. Gene rearrangements in mitogenomes are useful in reconstruction of Brachyuran phylogeny^19^. In the present study, we sequenced the complete mitogenome of *C*. *sinensis* with the aim of elucidating its evolutionary status and rearrangement information by comparing it with complete Brachyuran mitogenomes available to date^20, 21^. This information may provide insights into phylogenetic rearrangement and enable phylogenetic analysis.

## Methods

### Sample and DNA Extraction

Adult specimens of *C*. *sinensis* were captured from Yancheng, Jiangsu province, China. Total genomic DNA was isolated from individual specimens using the Aidlab Genomic DNA Extraction Kit (Beijing, China). All procedures were completed following the manufacturer’s instructions. The complete mitogenome was amplified from the DNA from one *C*. *sinensis* crab.

### PCR Amplification and Sequencing

The complete mitogenome was obtained using a combination of conventional PCR and long PCR to amplify overlapping fragments spanning the whole mitogenome. Universal and specific primers were designed based on the conserved nucleotide sequences of known mitochondrial sequences in Brachyura (Table 1) and synthesized by Beijing Sunbiotech^22–26^. The fragments were amplified using Aidlab Red Taq (Beijing, China) according to the manufacturer’s instructions. All amplifications were performed on an Eppendorf Mastercycler and Mastercycler gradient in 50 µl reaction volumes with 5 µl 10 × Taq Buffer (Mg^2+^) (Aidlab), 4 µl of dNTPs (2.5 mM, Aidlab), 2 µl of each primer (10 µM), 2 µl of DNA temple (~30 ng), 34.5 µl ddH_2_O, and 0.5 µl Red Taq DNA polymerase (5U, Aidlab). PCR was performed using the following procedure: 94 °C for 3 min; followed by 40 cycles of 30 s at 94 °C, annealing for 35 s at 48–56 °C (depending on primer combination), and elongation at 72 °C for 30 s to 4 min (depending on the fragment length); and final extension at 72 °C for 10 min. The PCR products were separated by agarose gel electrophoresis (1% w/v) and purified using a DNA gel extraction kit (Transgen, Beijing, China). The purified products were then ligated into the T-vector (Sangon, Shanghai, China) and sequenced.

**Table 1:** Primers used in this study.

Primer	Sequence (5′-3′)	annealing temperature	Location
F1	GGTCAACAAATCATAAAGATATTGG	55 °C	*cox1*
R1	TAAACTTCAGGGTGACCAAAAAATCA		*cox1*
F2	TAGTWATHAANGGHCTACGVTGRGG	50 °C	*cox3*
R2	AAGTCCRTGRAAYCCDGTDGCHAC		*cox3*
F3	TATGTGGDWTWCCTTTTWTAGCDGG	48 °C	*nad5*
R3	ATHTCAAGMTAARCHAGCHCCHCC		*nad5*
F4	GTGCCAGCCGCCGCGGTTA	52 °C	*rrnS*
R4	ATGCACTTTCCAGTACATCTA		*rrnS*
F5	CCCACGCAGGAGCTTCAGTAG	56 °C	*cox1*-*cox3*
R5	AGTCTTTGGATTGCTTGGTTGTG		*cox1*-*cox3*
F6	TTCCCCTTTTAAATACAACTA	56 °C	*cox3*-*nad5*
R6	GCTAATGCAGGGATACTAAC		*cox3*-*nad5*
F7	GCAGGTATCAAGCAGAAAAAG	56 °C	*nad5*-*rrnS*
R7	TTTAAAAATTTGGCGGTGAT		*nad5*-*rrnS*
F8	ATCAAATCCTCCTTCATAATA	56 °C	*rrnS*-*cox1*
R8	GCAGCAGCTAGAGGAGGATAAA		*rrnS*-*cox1*

### Complete Mitogenome Analysis

The graphical map of the complete mitogenome was drawn using the online mitochondrial visualization tool mtviz^27^. The secondary cloverleaf structure and anticodon of transfer RNAs were identified using the tRNA-scan SE webserver^28^. Codon usage and the nucleotide composition of the mitogenome were determined using MEGA6. The sequences of 29 Brachyura species and *Alpheus distinguendus* were aligned using MAFFT^29^.

### Phylogenetic Analysis

Twenty-eight complete Brachyura mitogenomes were downloaded from GenBank (https://www.ncbi.nlm.nih.gov/genbank/). In addition, the mitogenome of *A*. *distinguendus* was downloaded from GenBank and used as an outgroup taxon. GenBank sequence information is shown in Table 2.

**Table 2:** List of Brachyura species analysed in this study with their GenBank accession numbers.

Species	Family	Size (bp)	Accession No.
***Clistocoeloma sinensis***	**Sesarmidae**	**15,706**	**KU589292**
*Sesarmops sinensis*	Sesarmidae	15,905	KR336554
*Helice latimera*	Varunidae	16,246	KU589291
*Pachygrapsus crassipes*	Grapsidae	15,652	KC878511
*Eriocheir japonica sinensis*	Varunidae	16,378	KM516908
*Eriocheir japonica hepuensis*	Varunidae	16,335	FJ455506
*Eriocheir japonica japonica*	Varunidae	16,352	FJ455505
*Xenograpsus testudinatus*	Xenograpsidae	15,798	EU727203
*Homologenus malayensis*	Homolidae	15,793	KJ612407
*Pseudocarcinus gigas*	Menippidae	15,515	AY562127
*Damithrax spinosissimus*	Mithracidae	15,817	KM405516
*Geothelphusa dehaani*	Potamidae	18,197	AB187570
*Portunus pelagicus*	Portunidae	16,157	KM977882
*Callinectes sapidus*	Portunidae	16,263	AY363392
*Portunus trituberculatus*	Portunidae	16,026	AB093006
*Portunus sanguinolentus*	Portunidae	16,024	KT438509
*Charybdis japonica*	Portunidae	15,738	FJ460517
*Scylla paramamosain*	Portunidae	15,824	JX457150
*Scylla olivacea*	Portunidae	15,723	FJ827760
*Scylla tranquebarica*	Portunidae	15,833	FJ827759
*Scylla serrata*	Portunidae	15,775	FJ827758
*Charybdis feriata*	Portunidae	15,660	KF386147
*Umalia orientalis*	Raninidae	15,466	KM365084
*Lyreidus brevifrons*	Raninidae	16,112	KM983394
*Gandalfus yunohana*	Bythograeidae	15,567	EU647222
*Gandalfus puia*	Bythograeidae	15,548	KR002727
*Austinograea alayseae*	Bythograeidae	15,620	JQ035660
*Austinograea rodriguezensis*	Bythograeidae	15,611	JQ035658
*Ilyoplax deschampsi*	Dotillidae	15,460	JF909979

The sequences were aligned with the mitochondrial sequences of closely related species. In order to remove the gaps in sequences, poorly aligned positions and divergent regions were removed using Gblocks^25^. Then, fasta sequences were converted to nex format sequences and phylip format sequences for Bayesian inference (BI) and Maximum likelihood (ML) analyses using online software (http://sequenceconversion.bugaco.com/converter/biology/sequences/fasta_to_phylip.php). We used DAMBE to detect the saturation status of the sequences^30^.

We determined the taxonomic status of *C*. *sinensis* within Brachyura by reconstructing the phylogenetic tree. Nucleotide sequences from 30 mitogenome PCGs were combined. The dataset was run using two inference methods: BI and ML analyses. The former was performed using Mrbayes v3.2.1^31^, while ML analysis was performed using raxmlGUI^32^. The nucleotide substitution model was selected using Akaike information criterion implemented in Mrmodeltest v2.3^33, 34^. The GTR+I+G model was the best model to examine nucleotide phylogenetic analysis and molecular evolution. BI and ML analyses were performed under the GTRCAT model with nucleotide alignment (NT dataset) of the 13 mitochondrial PCGs. ML analyses were performed on 1000 bootstrapped datasets. The BI analysis ran as 4 simultaneous MCMC chains for 10,000,000 generations, sampled every 100 generations, and a burn-in of 5000 generations was used. The average standard deviation of split frequencies was less than 0.01, and the effective sample size determined using tracer v1.6 exceeded 200. These two findings indicate that our data was convergent. The resulting phylogenetic trees were visualized using FigTree v1.4.2.

## Results and Discussion

### Genome Structure and Organization

**Figure 1:**
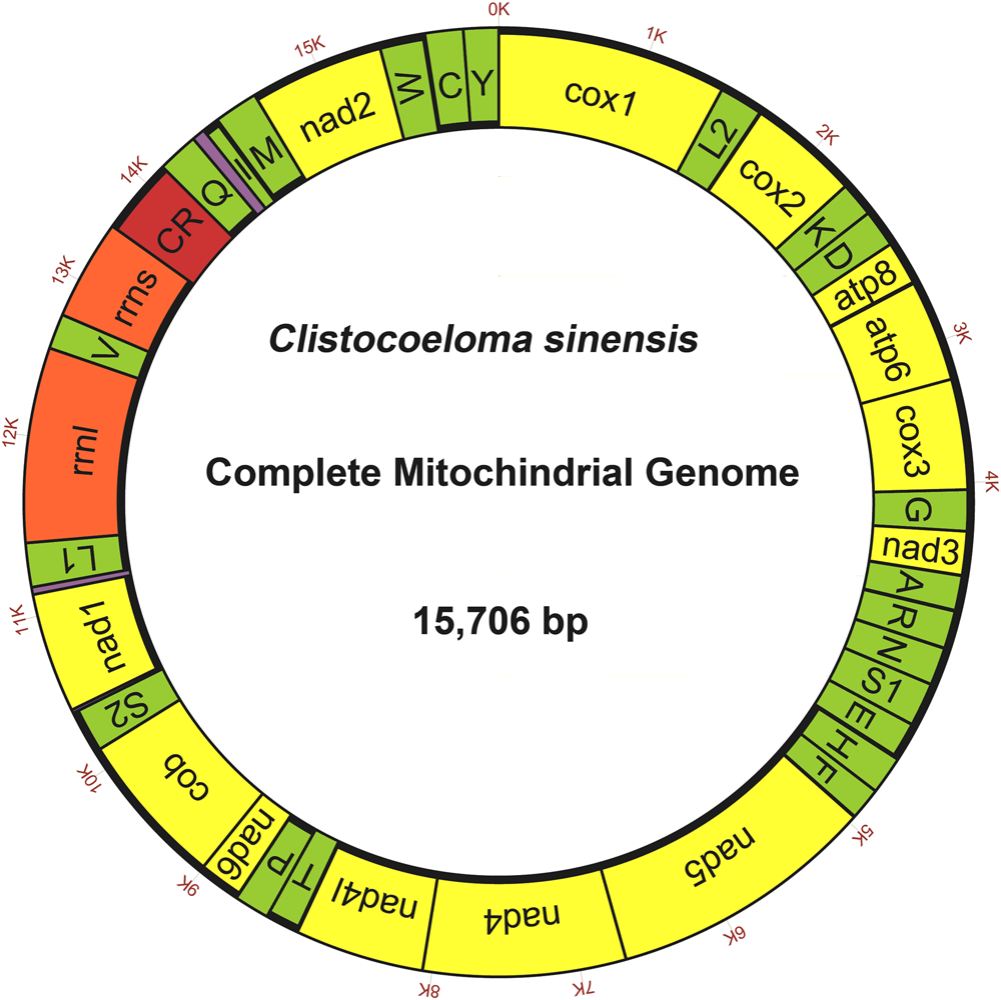
Graphical map of the mitogenome of *Clistocoeloma sinensis*. Protein-coding and ribosomal RNA genes are shown using standard abbreviations. Genes for transfer RNAs are abbreviated using a single letter. S1 = AGN, S2 = UCN, L1 = CUN, L2 = UUR. CR = control region. The 13 protein-coding genes are yellow, tRNAs are green, rRNAs are red, and CRs are dark red.

The mitogenome of *C*. *sinensis* is 15,706 bp long, and its gene content is same as that most known Brachyura: 13 PCGs, 2 rRNA genes, and 22 tRNA genes plus CR (Table 3 and Fig. 1). Twenty-three genes are coded on the J strand and the remaining 14 genes are transcribed on the N strand. It has been deposited in GenBank under accession number KU589292. The genome composition (A: 37.1%, T: 38.6%, C: 14.9%, G: 9.4%) shows a strong A+T bias, which account for 75.7% of the bases, and exhibits a negative AT skew ([A − T]/[A+T] = −0.020) and GC skew ([G − C]/[G+C] = −0.228). The A+T skew of other previously sequenced Brachyura mitogenomes ranged from −0.080 (*Pachygrapsus crassipes*) to 0.040 (*Homologenus malayensis*), while the G+C skew ranged from −0.341 (*Austinograea rodriguezensis*, *Geothelphusa dehaani*) to −0.219 (*Portunus pelagicus*) (Table 4). However, different regions have different A+T contents. The CR had the highest A+T content (82.9%), whereas the PCG region had the lowest A+T content (74.2%) (Table 5).

**Table 3:** Summary of *Clistocoeloma sinensis* mitogenome.

Gene	Direction	Location	Size	Intergenic nucleotides	Anticodon	Start codon	Stop codon
*cox1*	F	1–1535	1535	0		ATG	TA
*trnL2*	F	1536–1601	66	6	TAA		
*cox2*	F	1608–2295	688	0		ATG	T
*trnK*	F	2296–2365	70	0	TTT		
*trnD*	F	2366–2433	68	0	GTC		
*atp8*	F	2434–2592	159	−7		ATG	TAA
*atp6*	F	2586–3259	674	0		ATT	TA
*cox3*	F	3260–4050	791	0		ATG	TA
*trnG*	F	4051–4115	65	0	TCC		
*nad3*	F	4116–4466	351	2		ATT	TAA
*trnA*	F	4469–4532	64	5	TGC		
*trnR*	F	4538–4601	64	2	TCG		
*trnN*	F	4604–4674	71	1	GTT		
*trnS1*	F	4676–4743	68	−1	TCT		
*trnE*	F	4743–4810	68	9	TTC		
*trnH*	R	4820–4886	67	0	GTG		
*trnF*	R	4887–4951	65	4	GAA		
*nad5*	R	4956–6686	1731	0		ATG	TAA
*nad4*	R	6687–8065	1379	0		ATG	TA
*nad4L*	R	8066–8361	296	7		ATG	A
*trnT*	F	8369–8434	66	0	TGT		
*trnP*	R	8435–8502	68	2	TGG		
*nad6*	F	8505–9008	504	0		ATT	TAA
*cob*	F	9009–10,143	1135	0		ATT	A
*trnS2*	F	10,144–10,212	69	18	TGA		
*nad1*	R	10,231–11,169	939	39		ATA	TAA
*trnL1*	R	11,209–11,276	68	0	TAG		
*rrnL*	R	11,277–12,612	1336	0			
*trnV*	R	12,613–12,685	73	0	TAC		
*rrnS*	R	12,686–13,517	832	0			
CR	—	13,518–14,201	684	0			
*trnQ*	R	14,202–14,269	68	70	TTG		
*trnI*	F	14,340–14,405	66	12	GAT		
*trnM*	F	14,418–14,487	70	0	CAT		
*nad2*	F	14,488–15,493	1006	0		ATG	T
*trnW*	F	15,494–15,562	69	11	TCA		
*trnC*	R	15,574–15,637	64	0	GCA		
*trnY*	R	15,638–15,706	69	—	GTA

**Table 4:** Composition and skewness of mitogenome in 29 Brachyura species.

species	Size (bp)	A %	G %	T %	C %	A+T %	A+T skew	G+C skew
***C***. ***sinensis***	**15,706**	**37.1**	**9.4**	**38.6**	**14.9**	**75.7**	**−0.020**	**−0.228**
*S*. *sinensis*	15,905	37.4	9.4	38.3	14.9	75.7	−0.012	−0.228
*H*. *latimera*	16,246	34.0	11.0	35.1	19.9	69.1	−0.017	−0.290
*G*. *puia*	15,548	35.1	10.3	34.8	19.8	69.9	0.006	−0.313
*P*. *sanguinolentus*	16,024	31.6	12.9	34.0	21.5	65.6	−0.037	−0.243
*E*. *j*. *sinensis*	16,378	35.2	10.8	36.4	17.6	71.6	−0.016	−0.243
*E*. *j*. *hepuensis*	16,335	35.1	10.8	36.4	17.7	71.5	−0.018	−0.245
*E*. *j*. *japonica*	16,352	35.2	10.7	36.5	17.7	71.7	−0.018	−0.245
*X*. *testudinatus*	15,798	36.7	9.3	37.2	16.8	73.9	−0.007	−0.286
*P*. *gigas*	15,515	35.0	10.8	35.5	18.7	70.5	−0.006	−0.268
*G*. *dehaani*	18,197	36.9	8.3	38.0	16.8	74.9	−0.014	−0.341
*L*. *brevifrons*	16,112	34.2	11.3	36.4	18.1	70.6	−0.031	−0.231
*C*. *sapidus*	16,263	34.2	11.1	34.9	19.8	69.1	−0.011	−0.279
*P*. *trituberculatus*	16,026	33.3	11.3	36.9	18.5	70.2	−0.051	−0.241
*H*. *malayensis*	15,793	37.3	10.0	34.4	18.3	71.7	0.040	−0.292
*C*. *japonica*	15,738	33.8	11.9	35.4	18.9	69.2	−0.024	−0.228
*S*. *paramamosain*	15,824	34.9	10.1	38.2	16.8	73.1	−0.045	−0.247
*U*. *orientalis*	15,466	33.1	11.8	34.9	20.2	68.0	−0.027	−0.262
*S*. *olivacea*	15,723	33.5	11.2	35.9	19.4	69.4	−0.035	−0.267
*S*. *tranquebarica*	15,833	35.0	9.8	38.7	16.5	73.7	−0.050	−0.258
*S*. *serrata*	15,775	34.5	10.4	38.0	17.1	72.5	−0.047	−0.242
*D*. *spinosissimus*	15,817	33.3	10.5	36.8	19.4	70.1	−0.050	−0.294
*C*. *feriata*	15,660	34.1	11.2	36.1	18.6	70.2	−0.028	−0.246
*G*. *yunohana*	15,567	34.3	10.8	35.6	19.3	69.9	−0.019	−0.281
*P*. *pelagicus*	16,157	33.7	12.2	35.0	19.1	68.8	−0.019	−0.219
*A*. *alayseae*	15,620	34.4	11.4	32.4	21.8	66.8	0.029	−0.316
*A*. *rodriguezensis*	15,611	35.3	10.3	33.5	20.9	68.8	0.025	−0.341
*P*. *crassipes*	15,652	30.5	12.7	35.8	21.0	66.3	−0.080	−0.245
*I*. *deschampsi*	15,460	34.1	10.7	35.5	19.7	69.6	−0.019	−0.294

**Table 5:** Composition and skewness of *Clistocoeloma sinensis* mitogenome.

nt %	PCGs	tRNAs	rRNAs	CR
A%	36.1	37.7	40.4	43.4
T%	38.1	38.5	39.8	39.5
C%	15.9	12.8	13.0	10.5
G%	9.9	11.0	6.8	6.6
A+T%	74.2	76.2	80.2	82.9
C+G%	25.8	23.8	19.8	17.1
AT-Skew	−0.026	−0.010	0.007	0.047
GC-skew	−0.233	−0.075	−0.313	−0.228

CR = control region.

### Protein-Coding Genes

**Figure 2:**
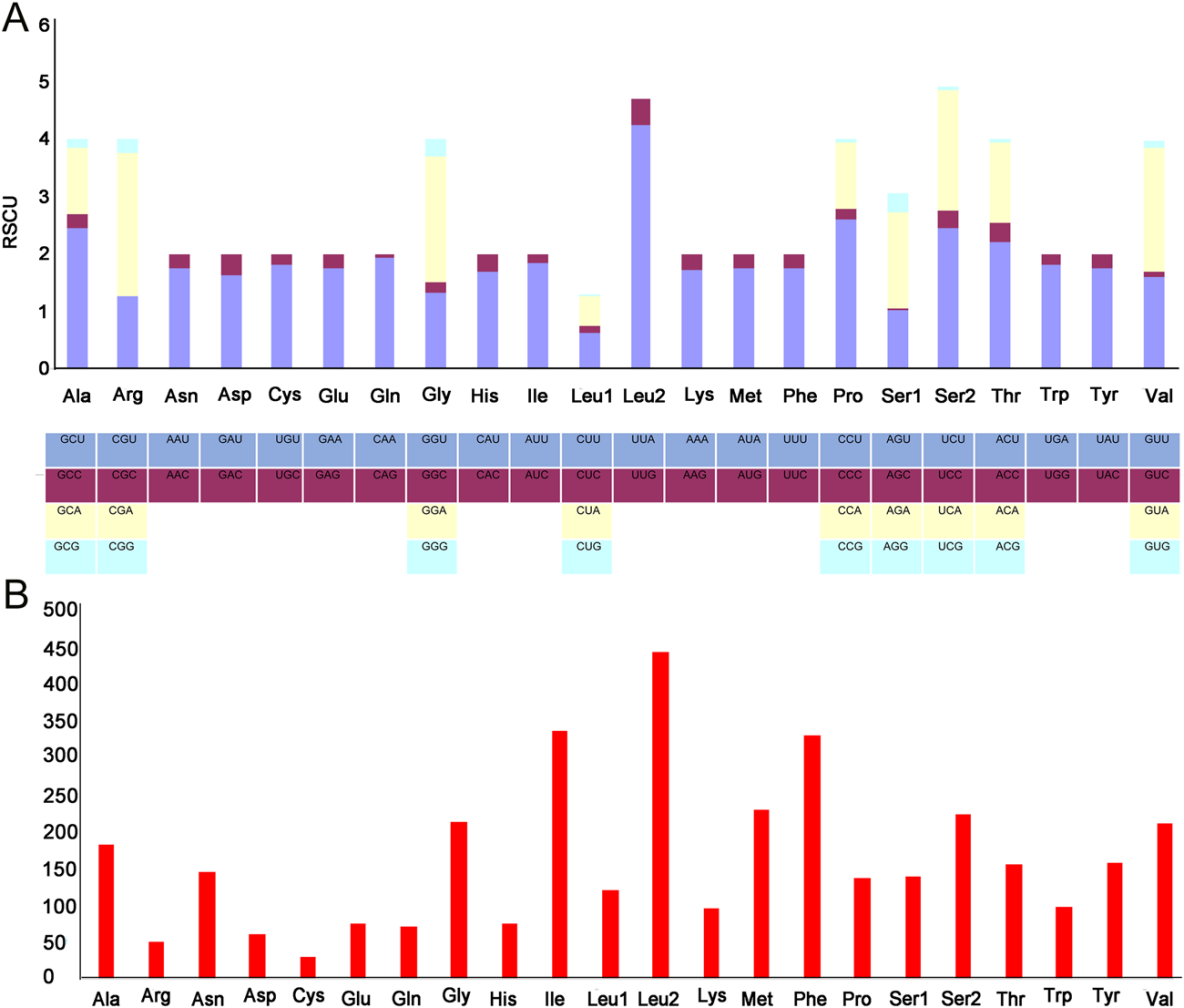
Relative synonymous codon usage in *Clistocoeloma sinensis* mtDNAs. Codon families are provided on the x axis (**A**). (**B**) Nucleotide composition conditions.

Among the 13 PCGs, 9 (*nad2*, *cox1*, *cox2*, *atp8*, *atp6*, *cox3*, *nad3*, *nad6*, and *cob*) were coded on the J strand, while the rest (*nad5*, *nad4*, *nad4L*, and *nad1*) were on the N strand. The 13 PCGs ranged in size from 159 to 1731 bp (Table 3). Their A+T content was 74.2% and AT skew was −0.026 (Table 5). The relative synonymous codon usage for *C*. *sinensis* at the third position is shown in Fig. 2. The usage of both two- and four-fold degenerate codons was biased toward the use of codons abundant in A or T (Table 6), which is consistent with other Brachyura species^35–37^.

**Table 6:** The codon number and relative synonymous codon usage in *Clistocoeloma sinensis* mitochondrial protein coding genes.

Codon	Count	RSCU	Codon	Count	RSCU	Codon	Count	RSCU	Codon	Count	RSCU
UUU(F)	291	1.75	UCU(S)	111	2.44	UAU(Y)	140	1.76	UGU(C)	27	1.8
UUC(F)	42	0.25	UCC(S)	14	0.31	UAC(Y)	19	0.24	UGC(C)	3	0.2
UUA(L)	401	4.24	UCA(S)	96	2.11	UAA(*)	8	2	UGA(W)	89	1.82
UUG(L)	45	0.48	UCG(S)	3	0.07	UAG(*)	0	0	UGG(W)	9	0.18
CUU(L)	60	0.63	CCU(P)	90	2.61	CAU(H)	64	1.68	CGU(R)	16	1.25
CUC(L)	10	0.11	CCC(P)	6	0.17	CAC(H)	12	0.32	CGC(R)	0	0
CUA(L)	49	0.52	CCA(P)	40	1.16	CAA(Q)	70	1.94	CGA(R)	32	2.51
CUG(L)	2	0.02	CCG(P)	2	0.06	CAG(Q)	2	0.06	CGG(R)	3	0.24
AUU(I)	312	1.85	ACU(T)	86	2.21	AAU(N)	128	1.74	AGU(S)	47	1.03
AUC(I)	26	0.15	ACC(T)	13	0.33	AAC(N)	19	0.26	AGC(S)	1	0.02
AUA(M)	203	1.76	ACA(T)	55	1.41	AAA(K)	82	1.71	AGA(S)	77	1.69
AUG(M)	28	0.24	ACG(T)	2	0.05	AAG(K)	14	0.29	AGG(S)	15	0.33
GUU(V)	85	1.6	GCU(A)	113	2.46	GAU(D)	50	1.64	GGU(G)	72	1.34
GUC(V)	5	0.09	GCC(A)	11	0.24	GAC(D)	11	0.36	GGC(G)	9	0.17
GUA(V)	115	2.17	GCA(A)	54	1.17	GAA(E)	66	1.74	GGA(G)	117	2.18
GUG(V)	7	0.13	GCG(A)	6	0.13	GAG(E)	10	0.26	GGG(G)	17	0.32

### Transfer RNAs, Ribosomal RNAs, and A+T-Rich Region

**Figure 3:**
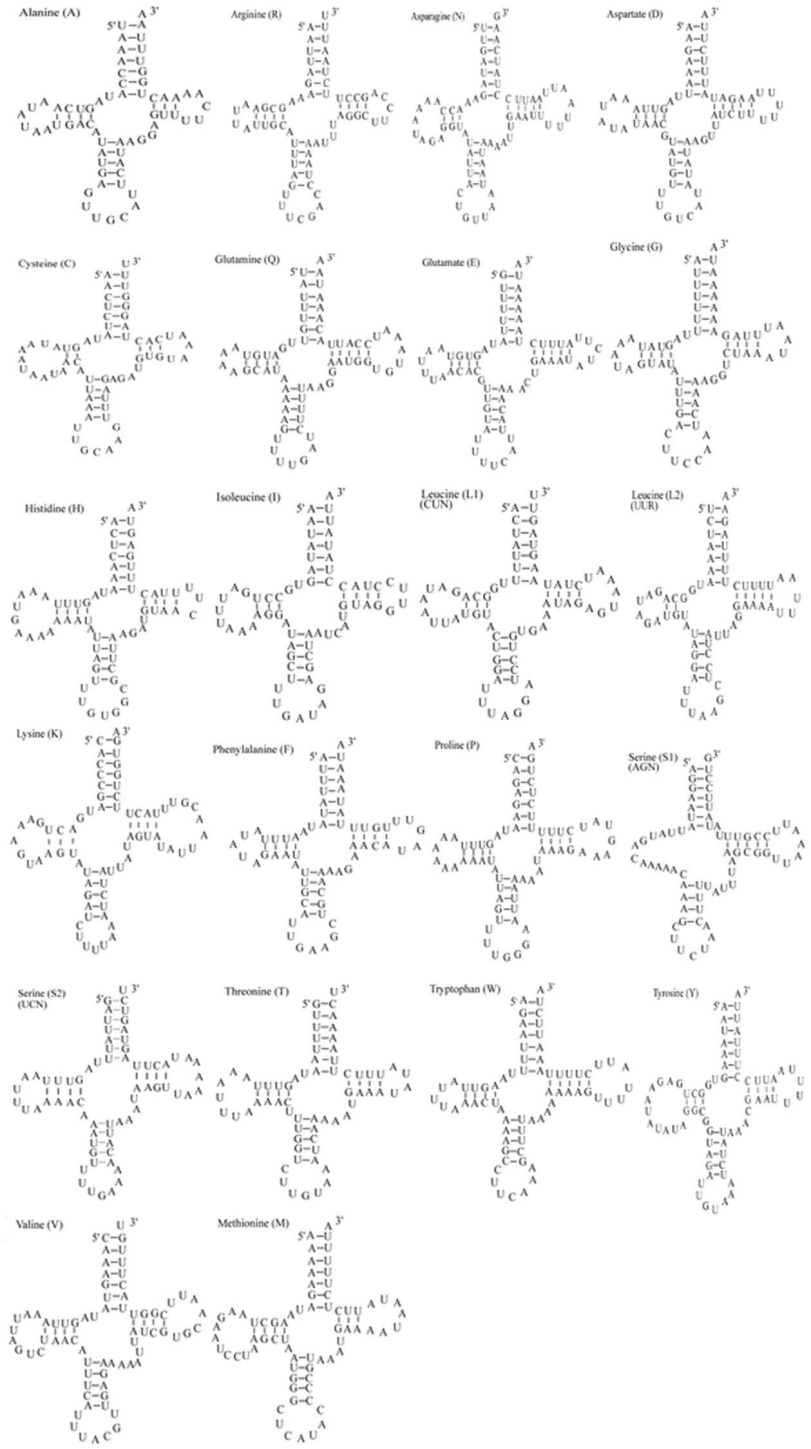
Secondary structures of the 22 transfer RNA genes of *Clistocoeloma sinensis*. The tRNAs are labelled with the abbreviations of their corresponding amino acids. Dashes (−) indicate Watson-Crick pairing.

Like most Brachyura mtDNA, the *C*. *sinensis* mitogenome contains a set of 22 tRNAs genes (Fig. 3), although this feature is not very well conserved in animal mtDNA. The tRNAs ranged in size from 64 to 73 bp and showed a strong A+T bias, as these bases accounted for 76.2% of the DNA. Further, they exhibited a negative AT skew (−0.010) (Table 5). Fourteen tRNA genes were present on the J strand and eight were on the N strand. All the tRNA genes had the typical cloverleaf structure, except for the *trnS1* gene, whose dihydroxyuridine arm was instead just a simple loop (Fig. 3). These features are common in most Brachyura mitogenomes^35–37^. The secondary cloverleaf structure of 18 tRNAs was examined using tRNA-scan SE; 4 tRNAs not detected by tRNAscan-SE were found in the unannotated regions by sequence similarity to the tRNAs of other crabs. The 2 rRNA genes with 80.2% total A+T content and positive AT skew (0.007) (Table 5) were located between *trnL1* and *trnV* and between *trnV* and CR. *rrnL* is 1336 bp while *rrnS* is 832 bp. The CR located between *rrnS* and *trnQ*, spans 684 bp. This region contains 82.9% AT nucleotides, with a positive AT skew (0.047) and negative GC skew (−0.228) (Table 5).

### Gene Arrangement

**Figure 4:**
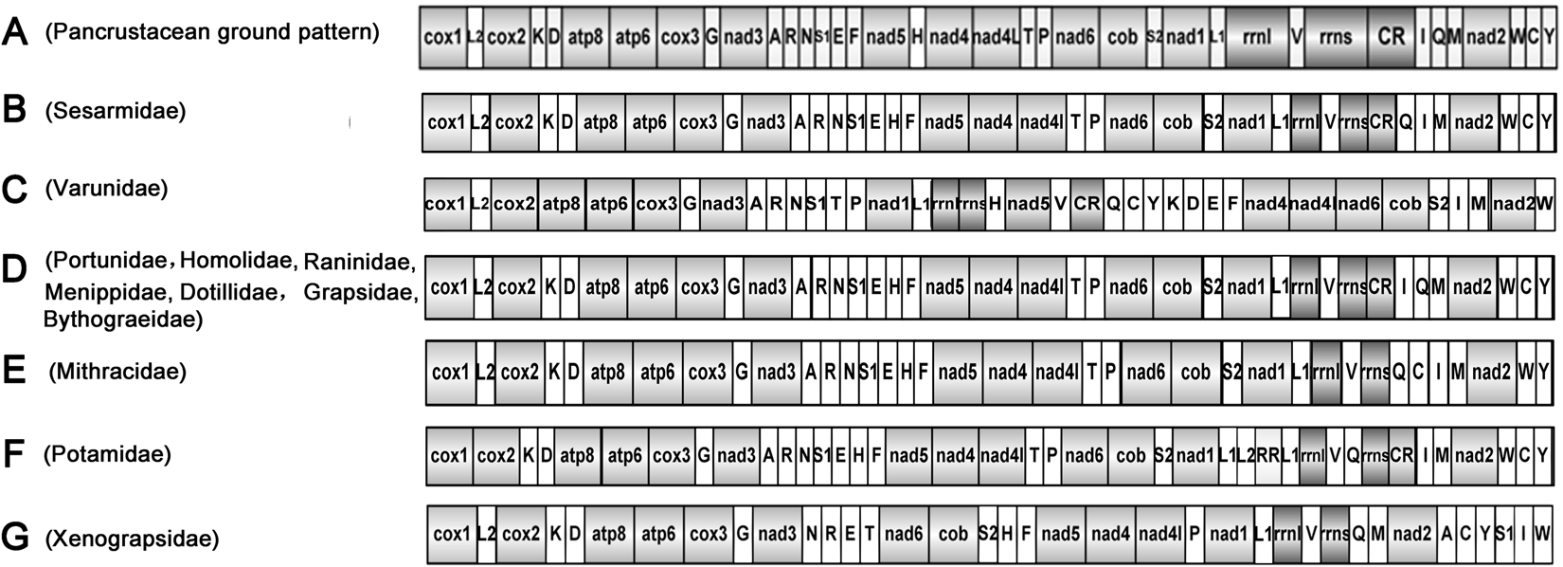
Linear representation of gene rearrangements of Brachyura mitogenomes. All genes are transcribed from left to right. tRNA genes are represented by the corresponding single-letter amino acid code. S1 = AGN, S2 = UCN, L1 = CUN, L2 = UUR. CR = control region. *rrnL* and *rrnS* are the large and small ribosomal RNA subunits.

Gene order within the complete mitogenome of *C*. *sinensis* is similar to the pancrustacean ground pattern^38–40^ (Fig. 4A), except for the translocation of *trnH*. Typically, the *trnH* gene is located between the *nad4* and *nad5* genes in the pancrustacean ground pattern, but in *C*. *sinensis*, it is between the *trnE* and *trnF* genes (Fig. 4B). This translocation was also observed in the mitogenomes of Brachyura crabs available in GenBank that were compared with the *C*. *sinensis* mitogenome. In addition, in the pancrustacean ground pattern, the tRNA gene order between the CR and *nad2* is *trnI*-*trnQ*-*trnM*. However, in *C*. *sinensis*, it is *trnQ*-*trnI*-*trnM* (Fig. 4B). The tRNA rearrangements are generally considered to be a consequence of tandem duplication of part of the mitogenome^41^. Similar non-coding sequences are present at the position of *trnI* originally occupied by the transposed *trnQ* in *C*. *sinensis*. Because these intergenic sequences have similar lengths to those of typical tRNA genes, they were presumed to be remnants of the *trnQ* gene and its boundary sequences^42^. The gene order of *C*. *sinensis* is identical to that of *S*. *sinensis* (Fig. 4B), which indicates that *C*. *sinensis* may belong to the group Sesarmidae of the superfamily Grapsoidea and that *C*. *sinensis* and *S*. *sinensis* probably belong to sister groups.

The gene sequences of Varunidae species (*Eriocheir japonica sinensis*, *E*. *j*. *hepuensis*, *E*. *j*. *japonica*, and *Helice latimera*) are identical (Fig. 4C). As shown in Fig. 4D, the order and orientation of genes in 7 families are uniform. The order of genes in *C*. *sinensis* sequences is different from that in the sequences of the mitogenomes of these 7 families because of the rearrangement of two tRNA genes between CR and *trnM*: the placement of genes between CR and *trnM* in *C*. *sinensis* is CR-*trnQ*-*trnI*-*trnM*, while that in the 7 families is CR-*trnI*-*trnQ*-*trnM*. In this case, tandem duplication of gene regions may be the most likely mechanism for mitochondrial gene rearrangement, which includes *trnI* and *trnQ*, followed by loss of supernumerary genes^43, 44^. Slipped-strand mispairing occurred first, followed by gene deletion^45^. Partial PCGs, tRNAs, and rRNAs of *Damithrax spinosissimus*, *G*. *dehaani*, and *Xenograpsus testudinatus* appear to be rearranged compared to *C*. *sinensis* (Fig. 4E–G).

### Phylogenetic analysis

**Figure 5:**
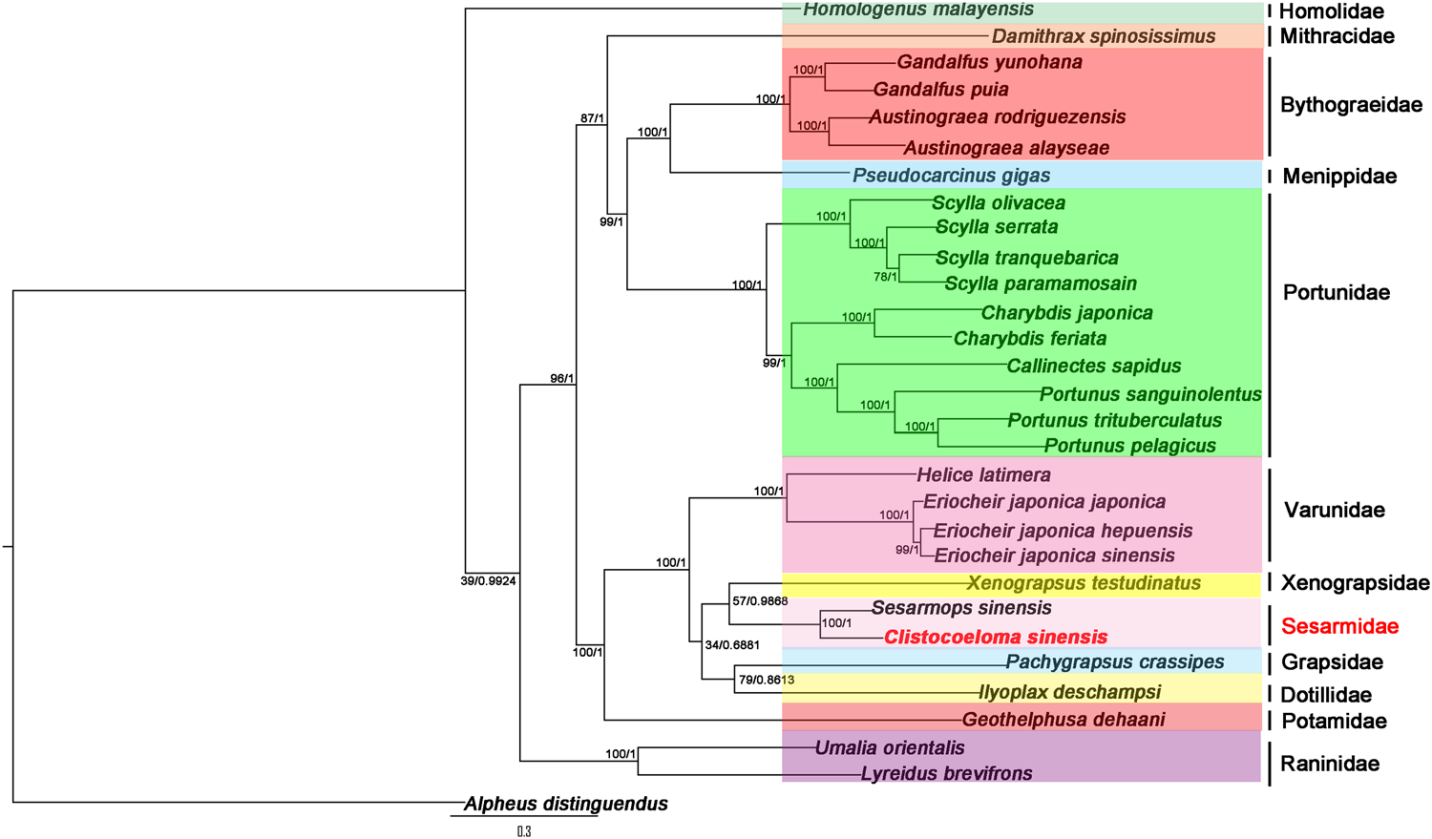
Inferred phylogenetic relationships among Brachyura based on nucleotide sequence of 13 mitochondrial PCGs using maximum likelihood (ML) and Bayesian inference (BI). *Alpheus distinguendus* was used as the outgroup. The bootstrap value (BP) and Bayesian posterior probability (BPP) of each node are shown as BP based on the NT dataset/BPP based on the NT dataset, 100/1.00.

Our analyses were based on the NT dataset in mitogenomes derived from 29 Brachyura species belonging to 12 families (Varunidae, Xenograpsidae, Homolidae, Menippidae, Mithracidae, Potamidae, Portunidae, Raninidae, Bythograeidae, Sesarmidae, Grapsidae, and Dotillidae). The data matrix (15,706 bp in all) was analysed using the model-based evolutionary methods of BI and ML analyses (Fig. 5). The ML and BI analyses of the dataset gave the same tree topology. It is obvious that *C*. *sinensis* and *S*. *sinensis* clustered in one branch in the phylogenetic tree with high nodal support values (Fig. 5), indicating that *C*. *sinensis* and *S*. *sinensis* have a sister group relationship. This result supported that *C*. *sinensis* belongs to Grapsoidea, Sesarmidae. From the phylogenetic tree, we found that *X*. *testudinatus* and two Sesarmidae species formed a group and showed close relationships. *X*. *testudinatus*, which was originally placed in Varunidae, has been transferred to its own family (Xenograpsidae)^21, 46^. Analysis of the nucleotide sequences of the 13 mitochondrial PCGs using BI and ML showed that *E*. *j*. *sinensis*, *E*. *j*. *hepuensis*, *E*. *j*. *japonica*, and *H*. *latimera* clustered together with high statistical support, showing that these species have a sister group relationship and belong to Grapsoidea, Varunidae. Our phylogenetic analysis indicated that Sesarmidae species, Xenograpsidae species and Varunidae species have close relationships^47^. In addition, *P*. *crassipes* belongs to Grapsoidea, Grapsidae^48^.

The phylogenetic position of *Ilyoplax deschampsi* is always within Grapsoidea^21, 47, 49, 50^. *I*. *deschampsi* belongs to the family Dotillidae, Ocypodoidea. The real phylogenetic position of *I*. *deschampsi* should be closer to the Grapsoidea species that shown in Fig. 5. Recent studies on the genus *Ucides* have also shown similar classification^51, 52^. *G*. *dehaani* belongs to Potamidae, Potamoidea^53^. However, the phylogenetic tree showed that Potamidae are associated closely with Varunidae, Grapsidae, Sesarmidae, Dotillidae, and Xenograpsidae. This result is in agreement to that inferred from 23 Brachyuran crabs, in which the author use the two mitogenomes^21^. Phylogenetic relationships between *I*. *deschampsi*, *G*. *dehaani* and Grapsoidea species need to be reconsidered by integrating more mitogenomic data. More mitogenomic data will also lead to a better overall understanding the phylogenetic relationships among Brachyuran crabs.

### Availability of data and materials

The data set supporting the results of this article is available at NCBI (KU589292).
